# A Case of Pneumonia in Its Third Stage; Death of the Patient, and Post Mortem Appearances

**Published:** 1853

**Authors:** E. A. Possy

**Affiliations:** Athens, Ky.


ARTICLE VI.—*A case of Pneumonia in its third stage; death of the patient, and post mortem appearances.* —By E. A. POSSY, M. D., of Athens, Ky.

HISTORY.—A young negro girl about thirteen years old, was taken, sometime last January, with pain in the chest, cough, dyspnœa, and a full vigorous pulse, indicating evidently the existence of inflammatory action going on in the chest. Dr. —— was sent for, and immediately resorted to medical means—what they were I was not able to learn. Suffice it to say, that bloodletting was not resorted to at all. On the contrary, it was, I suppose, prescribed agreeably to their respective system. The reader will not therefore be at a loss to imagine the great extent of injury inflicted upon the lungs, by allowing the inflammation to run its own course into hepatization and suppuration, without receiving, at its onset, a decided check by means of sanguineous depletions. In this primary stage of pneumonia, we know that the lung or lungs, if both be affected, are in a perfect state of engorgement, infiltrated with a frothy, sanguineous serosity, in considerable quantity. And that it is only and mainly by general and local bloodletting we can possibly unload those congested organs, and overcome the high vascular excitement prevailing at the same time. Yet bloodletting has been condemned; and in certain writings, the excellent use of the lancet has been brought into great discredit. And I remark; how are we to treat acute inflammatory affections, without the employment of that great and beloved instrument, the lancet? The lancet is the most important, and the most necessary; without it we are compelled to surrender instead of conquering. Pneumonia, in its first stage, presents a pure inflammation, occupying, or making haste to occupy, not only a few square inches, but many square inches of an organ or organs, whose functions are most highly important to life. It behooves us, then, in such an important case to resort to the most active and powerful antiphlogistics, and among such, every well educated and experienced practitioner resorts to venesection as the sheet anchor. And I reiterate here that nothing but abstractions of blood can exercise an immediate and powerful influence upon the lungs as well as the circulation, and reduce the momentum of the blood to such a degree of moderation, that with proper auxiliaries, we can but seldom fail to establish a resolution of the inflammation. If there is any inflammation in which bold, daring, and judicious venesection may be employed, it is this. Bearing, at the same time, in mind, that when the lancet can do no more, that we have two other substitutes in tartarized antimony and mercury. Tartarized antimony I deem best adapted to the first degree—that of engorgement; the mercurial plan to that of the second—that of hepatization. The inflammatory action not having been subdued in the first stage, hastened with formidable speed and rapidity to the second, and from thence to the third stage. The latter, in which I was called, the patient having been given out two weeks before, by the attending physician, as a hopeless case of phthisis pulmonalis. But on examination of the patient, I pronounced it a case of double pneumonia of the inferior lobes of the lungs—a diagnosis, as you readily perceive, diametrically opposed to that of the attending physician in question. And being fully convinced of the fact of my assertion, I could not withhold expressing an opinion, which was afterwards amply corroborated by the post mortem examination. The physical signs on which I founded my diagnosis were these: on percussion, I found the chest to elicit a clear resonant sound at the upper part, and upon auscultation, I discovered that the respiratory murmur was only audible in the superior part of the chest, signifying at once that the superior lobes were unaffected. And on the contrary, I found universal dullness over the lower part of the chest, and the respiratory murmur wholly inaudible, indicating that the main and chief mischief existed in the inferior lobes. I had also other important symptoms, such as bronchial respiration, expansion of the upper sides of the chest. I was told also that she had had rigors, and on a second auscultation, a peculiar muco-crepitating rhonceus was heard—this with the muco-purulent expectorations—were to me undoubted signs of pneumonia in its third stage—that of suppuration. Hav-been called at this late period, to minister upon this case, it was incumbent upon me as a practitioner of medicine, after having thoroughly explored the thorax of the patient, to give an opinion in regard to a favorable oı unfavorable termination of the case. The reader will readily imagine from the circumstances above, what would have been the prognosis of an experienced observer in such a case as this; evidently the most unfavorable. But notwithstanding the great prostration and depression of the vital powers, and the frightful emaciation of the patient, I resolved to comply with the old and good adage (*Nil desperandum*) never to despair, and had recourse immediately to certain medical means, if not with the object of affecting a cure, at least to alleviate considerably the sufferings of the patient. Agreeably to this, I resorted to tonics and stimulants without deriving any material benefit, save a palliation of the sufferings. The prostration and depression were too great, the general emaciation too frightful, and the texture of the lungs too far destroyed and involved in the process of suppuration, to do any good; and in fact the nervous and vascular systems had received a too-powerful shock to be benefited by the exhibition of remedies. Remedies were useless and powerless, and the patient bound to sink rapidly into the hands of death, in despite of the external administration of cordials and opiates—the topical application—and of counter-irritants. A few days after I saw the patient, she died; and being more than anxious to confirm my diagnosis, I obtained permission to perform the post mortem examination, which revealed to me pathological evidences which I had already anticipated. On the examination of the lungs, I discovered that by compression between the fingers, crepitation of the upper lobes was produced. They partially sank in water, owing to the upper lobes not being solidified; had they also been solidified as well as the lower lobes, they would have certainly sunk, as proved by the sinking of the lower lobes. Externally, the lungs presented a red color; internally they universally presented a characteristic grayish color, the pulmonary tissue dense, compact, and impervious to the air. When cut through, and slightly compressed, without crumbling it, liquid pus appeared on the surface, issuing from the grayish granulation; which were to me sufficient pathological evidences to convince the most incredulous observer, that it was pneumonia in its third stage—that of gray softening and not of phthisis pulmonalis. The diagnosis between pneumonia and pulmonary consumption is very obvious, and the post mortem examination shows evidently the existence of tubercular matter in lungs characterized by yellowish round masses—or by small gray and yellow granulations, sometimes in very large quantities—and by the tubercular infiltration, the morbid matter being extensively diffused throughout the substance of the lungs, and not in agglomerated masses, as it is in pneumonia. And we know too that the upper lobes are more frequently attacked, and the upper lobe of the left lung most frequently Pneumonia, the lower lobes of the right lung, having submitted the post mortem examination of this case, with all its consequences. The reader cannot but be convinced that it was a case of pneumonia in its third stage—that of suppuration.

